# Exploring the One Health Paradigm in Male Breast Cancer

**DOI:** 10.1007/s10911-024-09560-6

**Published:** 2024-04-04

**Authors:** Kirsty Luo-Yng Tay, George Cowan, Subarnarekha Chatterji, Giulia Conti, Valerie Speirs

**Affiliations:** 1 School of Medicine, Medical Sciences and Nutrition, University of Aberdeen, Aberdeen, AB25 2ZD UK; 2 Aberdeen Cancer Centre, Aberdeen, UK; 3 Department of Molecular Medicine, Sapienza University of Rome, Rome, Italy

**Keywords:** Male breast cancer, One health, Canines, Felines, Primates

## Abstract

How cancer patterns in humans compare to those of other species remains largely unknown and there is an even bigger knowledge gap for rare cancers like male breast cancer. One Health is a convergence of human and animal healthcare that encourages cross-pollination of medical research uniting human and veterinary medicine. Recognising that breast cancer occurs spontaneously in other male species (e.g. primates, canines, felines), and knowing that no laboratory models exist for male breast cancer, which limits our ability to perform functional studies, we explored the feasibility of applying One Health to breast cancer in men by conducting a narrative review of the topic. Spontaneous development of breast cancer was reported in captive male primates and in companion canines and felines. Some parallels in tumour biology of human male breast cancer with canines and primates were found. The age distribution, pattern of biomarker expression and metastasis were similar, with mammary tumours typically detected after two-thirds of average lifespan. However, instances of triple negative and inflammatory breast cancer, which are rarely observed in human male breast cancer, were found in canines and histological classification was inconsistent between species. These disparities need redressing to enable full exploration of the One Health paradigm in rare cancers.

**Supplementary Information:**

The online version contains supplementary material available at 10.1007/s10911-024-09560-6.

## Introduction

**Fig. 1:**
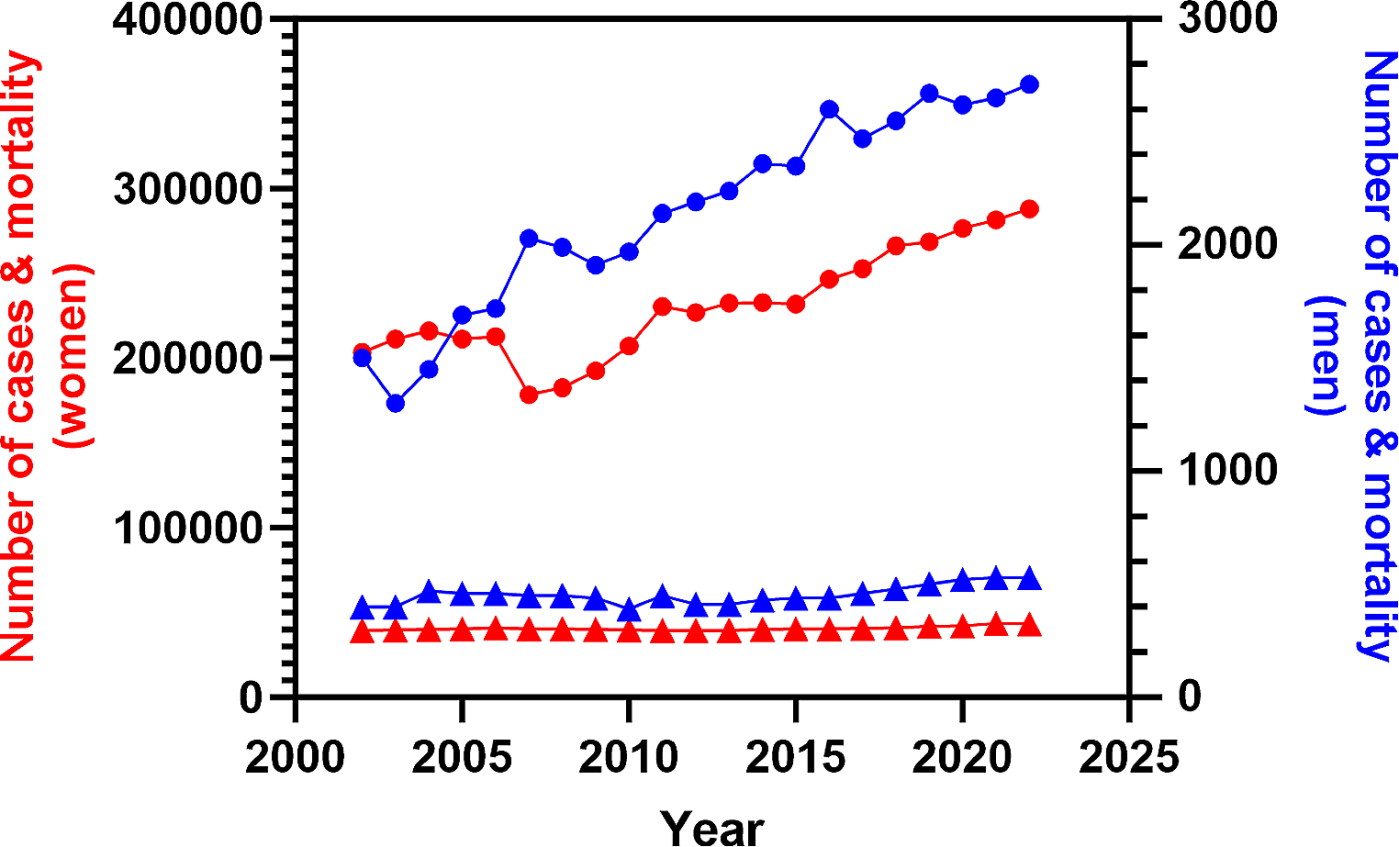
Incidence (circles) and mortality (triangles) trends of male (blue line, right axis)) and female (red line; left axis) breast cancer in the United States from 2002 to 2022. Graph constructed using data reported in the annual ‘Cancer Statistics’ publication by CA: A Cancer Journal for Clinicians [4, 5]

Some 400 men in the UK and an estimated 2800 in the US receive a breast cancer (BC) diagnosis each year, which is considerably fewer than the 56 000 and 297 000 women who are diagnosed, respectively, accounting for less than 1% of all breast cancer diagnoses worldwide [1]. However, as average lifespan increases, there has been a noticeable rise in male BC prevalence [2, 3], reflected in age-standardised data collected by the American Cancer Society over the last two decades [4, 5]. Indeed, a compilation of these data from 2002 to 2022 shows that the number of cases of male BC has increased from 1500 to 2710 per 100,000 persons (Fig. 1).

As BC in men is rare, it has been historically difficult to collect sufficient samples for studies to extend beyond anecdotal findings. This gap has been addressed through the establishment of the Male Breast Cancer Consortium and International Male Breast Cancer Program [6, 7], where large numbers of cases have been collected and centralised, allowing more rigorous characterisation. The focus of these consortia has been to collect formalin-fixed paraffin embedded tissues, predominantly. These have shown that male BC more often expresses estrogen (ER), progesterone (PR) and androgen receptors (AR), with a lower likelihood of human epidermal growth factor 2 (HER2) overexpression [6, 8–10]. Invasive ductal carcinoma accounts for approximately 83% of all male BC cases whereas lobular cancer is infrequent in men due to the absence of lobule development [11]. Other forms, including cribriform, intraductal papillary, mucinous, micropapillary, and papillary phenotypes, have also been reported in men, with papillary subtype showing a higher prevalence compared to women [8]. The principal molecular subtype in male BC is luminal A, with lower frequencies of luminal B and basal-like subtypes, including triple negative [6, 8].

Genomics and transcriptomics studies, indicate further differences between male and female BC, leading to the identification of two exclusive subgroups in men: luminal M1 and M2, distinguished by their unique tumour characteristics [12] and independent of the PAM50 subtypes in women. These findings, along with others, point towards male BC possessing a distinct genetic, histological, and receptor expression profile [6, 13–15].

While differences that separate male and female BC are being uncovered, the scarcity of male cases means that pre-clinical laboratory models are lacking. Numerous mouse models exist to study female BC [16], however male mice have only vestigial mammary tissue and lack nipples [17, 18], limiting their use as models. Recently, an ovine model has been proposed as a useful comparative model to understand the role of the microenvironment of the male mammary gland [19]. The “One Health” concept serves as a useful paradigm that leverages the shared knowledge of anatomy, pathology and physiology between human and veterinary medicine [20]. This approach highlights the potential of cross-species collaboration which has been demonstrated in various research studies [21, 22]. One Health was applied to investigate naturally-occurring osteoarthritis in canines to better understand the pathogenesis, molecular mechanisms, and potential treatment strategies relevant to humans [23]. Another study explored the similarities between humans and animals, particularly canines and equines, in tendon structure, function, and pathology [24] The One Health philosophy has also been proposed as a way of accelerating our understanding of understanding the pathology, diagnosis and treatment of mammary cancer across species [25, 26].

The translatability of spontaneously developed mammary cancer in non-human mammals, notably canines and felines is garnering interest [27–30] with previous research showing that spontaneous animal tumours are viable translational models in female BC [31, 32]. As companion animals often share the same environment as their owners and adopt some of their traits, they may develop the same comorbidities, including physical inactivity, a recognised risk factor for BC development [33, 34]. This has come on the back of detailed investigations into the genomic landscape of canine cancers which now exist alongside various analytical tools and images on public repositories [35]. However, there are no investigations into the applicability of using spontaneously developed mammary tumour in non-human male animals to model BC in men. Therefore, the aim of this work was to review the literature on spontaneously developed BC in non-human male mammals to determine its frequency and similarity to BC in male humans with a view to using these as pre-clinical models, which do not exist currently for male BC.

## Literature Searching

For this narrative review, a comprehensive search of databases, Ovid MEDLINE^®^ (1946 to June 12, 2023), Embase Classic + Embase (1947 to June 12, 2023), CAB Abstracts (1973 to June 12, 2023), All EBM reviews, Web of Science Core Collection, and SCOPUS, was conducted without restrictions on year or language. The search strategy involved compiling synonymous search terms for “breast,” “cancer,” and “men,” which were then combined with a comprehensive list of common animals from major mammalian classes.

## Non-human Primates

Non-human primates share over 90% of DNA with humans. This genetic similarity offers comparability in research findings and explains why they are sometimes used in medical studies. Scientists made significant discoveries about diseases, disorders, prevention, and treatments for both humans and animals, tying in the ‘one approach’ technique long before One Health was touted and applying results from studies on non-human primates to humans. A prime example of this is the discovery of insulin over a century ago [36] which laid the foundation for this type of approach, demonstrating a compelling basis for developing novel treatment modalities and harnessing comparative research efforts across species. More recently, the relative value of using non-human primates to model BC has been reviewed [37].

Case reports of BC detected in a handful of male non-human primates in captivity during routine physical examinations have been described. Species include rhesus macaque (*Macaca mulatta*), squirrel monkey (*Saimiri sciureus*), and orangutan (*Pongo pygmaeus*) [38–40]. A mammary lesion was found in the rhesus macaque, diagnosed as a spontaneous ductal carcinoma in situ (DCIS), which is a pre-invasive lesion [38]. In women DCIS is picked up frequently as one of the unintended consequences of national breast screening programmes [41]. However, DCIS is rarely seen in men, who typically present with more advanced disease [42]. In the squirrel monkey, an elevated subcutaneous nodule was surgically removed by wide excisional biopsy and diagnosed as an adenocarcinoma. No further treatment was given, but when the monkey was euthanised 18 months later for a comorbidity, a positive lymph node was identified [40]. The tumour resected from the male orangutan was subjected to the same diagnostic work up as human BC and immunohistochemistry revealed the expression of hormone receptors. The animal received tamoxifen at the same dose as humans, but the disease progressed. Switching to anastrozole, an aromatase inhibitor, slowed progression and the animal died from unrelated causes 4.5 years after diagnosis [39]. This treatment plan mirrored that recommended by ASCO for management of human male BC [43]. In another primate, BC was detected as an incidental finding in an autopsy of a male Humboldt’s white-fronted capuchin (*Cebus albifrons*) who died after being attacked by a male baboon [44]. A hormone receptor positive grade 2 tubular carcinoma, which is rare in men [8] was diagnosed.

## Non-primate Male Species

Forty papers reported mammary tumours in non-human non-primate male species, with a higher prevalence in canines and felines. A summary of species and age distribution is shown in Table 1. Mammary tumours typically occurred after two-thirds of the average lifespan. Canines comprised most cases, across 33 different breeds, with Cocker Spaniel (*n* = 14), German Shepherd (*n* = 6), and Dachshund (*n* = 6) most commonly reported, suggesting possible breed associations [45–57]. Indeed, previous studies have noted a preponderance of mammary tumours in these breeds among female canines [58]. Felines were the second most represented species, mainly in Domestic short-hairs, followed by four Siamese, three Domestic long-hair, and one Persian cat [59–62]. A single case of a simple ductal papilloma was reported in a captive maned wolf (*Chrysocyon brachyurus*) [63]. Papillary subtypes are seen more frequently in human male than in female BC [8]. Canines, felines and the lupine were diagnosed at an older age, median 10 years (range 1–15, canine) and median 11.5 years (range 3.5–19, feline).

**Table 1:** Age distribution across non-primate species diagnosed with male mammary tumours

Taxonomic Group/Species	Median age (sample size)	Range (sample size)
Canine [45–57, 64–70]	10 years (*n* = 76)	1–15 years (*n* = 84)
Feline [59, 61, 62, 71]	11.5 years (*n* = 4)	3.5–19 years (*n* = 43)
Rat [72, 73]	18 months (*n* = 3)	8.5–24 months (*n* = 3)
Guinea pig [74–79]	4 years (*n* = 7)	1.25–5.33 years (*n* = 7)
Rabbit [80]	7 years (*n* = 1)	NA
Wolf [63]	9 years (*n* = 1)	NA

NA, data not available or unreported

Of the 84 reported cases of male mammary tumours in canines, 29 were malignant (Table S1). This classification was based on published guidelines for the histological classification for canine mammary tumours [81]. The remaining benign neoplasms included simple adenomas, fibroadenomas, intraductal papilloma, and benign mixed tumours. In felines, all mammary neoplasms identified were reported as malignant, except one case of cystic adenoma papilliferum. Most of the subtypes diagnosed were aggressive, with two distinct histological diagnoses observed: metastatic ductal adenocarcinoma and invasive micropapillary carcinoma [62, 71]. Thirty-nine cases reported in one paper were described as “mammary adenocarcinoma” without detailed histological classification [61]. However, mammary cancers in felines tend to lack heterogeneity and are usually more aggressive [82]. Indeed, felines had the highest proportion of tumour-related deaths among all species described in this review. Formal classifications for mammary tumours in domestic species, including canines and felines, do exist [83] and whilst adopted widely, this may not always be universal.

Biomarkers used to classify human BC have been described in non-primate male animals (Table 2).

**Table 2:** Status of common immunohistochemical markers reported in cases of non-primate male mammary tumours

Paper	Species (no of cases)	Immunohistochemical Marker Expression			
		ER	PR	HER2	Ki-67
Arias et al. [48]*	Dog (1)	Positive	ND	ND	ND
Kwon et al. [55]*	Dog (1)	Positive	Positive	ND	ND
Saba et al. [53]	Dog (8)	Positive	Positive	ND	ND
Machado et al. [45]*	Dog (2)	Negative	Negative	Negative	ND
Mamom et al. [57]	Dog (2)	Negative	Positive	Negative	Positive
Gopal et al. [84]*	Dog (2)	Negative	Negative	Negative	ND
Saranya et al. [85]*	Dog (1)	ND	ND	Positive	Positive
Gregorio et al. [62]*	Cat (1)	ND	ND	ND	Positive
Cassali et al. [63]	Maned Wolf (1)	Positive	ND	ND	ND

ND, not done; *, malignant tumour

While complete data for all cases was patchy, 19 cases across 9 papers documented the three key biomarkers used for reporting human BC (ER, PR, HER2), and in some cases, Ki-67. These cases were predominantly canine [45, 48, 53, 55, 57, 84–86], with one feline [62] and one lupine [63]. Triple-negative mammary cancer was observed in three male dogs, an unusual finding in human males, where BC is predominately hormone receptor positive [8]. However, ER-positivity was reflected in most of the cases where ER was examined. Despite this, endocrine therapy (tamoxifen) is not recommended in canines due to adverse side effects [87]. It is worth noting that in veterinary medicine and basic sciences, immunohistochemistry is not always conducted to the same rigorous standards required for clinical reporting in humans. This would need to be addressed were a One Health approach to be implemented.

## Other Species

Among male rodents, eight out of ten reported BC cases were malignant tumours. Three were in rats (*Rattus norvegicus*); two in pets and one in a Wistar rat from a breeding colony, comprising two ductal mammary carcinoma and one papillary mammary carcinoma [72, 73]. A handful have been reported in male guinea pigs (*Cavia porcellus*) including invasive papillary carcinoma, solid simple mammary carcinoma, papillary cystadenocarcinoma, or benign tumours [74–79]. One case, a solid anaplastic adenocarcinoma, was reported in an intact male pet rabbit (*Oryctolagus cuniculus*) aged 7 years [80].

Although the focus of this paper was to explore the spontaneous development of male BC in non-human males, it is known that spontaneous tumour initiation can occur in oncogene-driven transgenic mice, typically MMTV-PyMT strains, which closely parallel human BC development and progression [88]. In a different transgenic strain FVB/N-Tg(MMTV-PyVT)634Mul/J (known as PyVT; [89]) mammary tumours developed spontaneously in male animals from 14 weeks of age which expressed ERα and -β, PR and HER2 [90]. HER2 is very rare in male BC [8], although ERβ has been reported [91]. Tumour burden was reduced following treatment with cisplatin but not paclitaxel or tamoxifen [90]. The latter finding is unusual as tamoxifen is typically first line choice for male BC [43]. Indeed, lack of response to tamoxifen in this transgenic mouse strain and its poor tolerance in canines [87] point to dissimilarities in physiology between species.

## Management and Outcomes

Animals tended to be diagnosed with larger tumours compared to the average 2.4 cm tumour size in human male BC [92] as shown in Table  S2. This discrepancy may arise as animals depend on their owners to notice the tumour, while in humans, palpable lumps would trigger suspicion. That said, BC in men is frequently diagnosed at a later stage due to a lack of awareness and stigma surrounding what is perceived by the stigma surrounding what is perceived by the public as a female cancer [93, 94]. Soft tissue metastases, but not the more common bone metastasis seen in male BC was observed in 8 animals. This aligns with previous studies highlighting a low incidence of bone metastasis in animals [95].

Surgery was performed in most canines (80/84), typically mastectomy and lumpectomy, like humans. Similarly, in felines all mammary tumours were managed surgically, with half of those unspecified. Lymph nodes were resected in two cases during radical mastectomy or lumpectomy [62, 71]. The specific surgical interventions for canine and felines were reported in Table 3.

**Table 3:** Frequency of surgical management in male mammary tumours in canines, felines, rodents, and primates

Surgical management	Canine	Feline	Rodents	Primates
Radical mastectomy	1 (1.25%) [45]	2 (4.65%) [61, 62]	0	0
Regional mastectomy	4 (5%) [48, 51, 53, 65]	2 (4.65%) [59, 60]	0	0
Simple mastectomy	7(8.75%) [47, 52, 55, 57]	8 (18.6%) [61]	1 (20%) [77]	0
Lumpectomy	16 (20%) [53, 56, 67–70, 84, 86, 96]	10 (23.3)	3 (60%) [72, 79]	1 (33.3%) [39]
Excisional biopsy	0	0	0	2 (66.6%) [38, 40]
Unspecified surgery	52 (65%) [45, 47, 49, 50, 54, 55, 57, 66, 97]	21 (48.8%) [61, 71]	1 (20%) [78]	0
**Sum of surgical management**	80	43	5	3
**% of cases receiving surgical management**	95%	100%	100%	100%

Post-operative chemotherapy with doxorubicin and cyclophosphamide was administered in two feline cases [61], again similar to humans. In canines, two simple mammary carcinoma cases received Cytocristin chemotherapy post-lumpectomy while two inflammatory mammary carcinomas were managed with analgesics and anti-inflammatories [46, 85, 96]. The cases of inflammatory breast cancer (IBC) presented like humans, with rapid onset of skin erythema and tumour emboli in the dermal lymphatics however chemotherapy is preferred in humans due to its aggressive nature [98]. This was reflected in the short lifespan of these canines’ post-diagnosis. The wolf with a simple ductal mammary papilloma received unspecified surgery [63]. In rodents, one rat underwent a simple mastectomy, while two received a lumpectomy with cisplatin electrochemotherapy [72, 73]. Lumpectomy with regional node removal was performed in one guinea pig [79].The rabbit with solid anaplastic carcinoma underwent excisional biopsy [80]. However, treatment protocols cannot readily be compared in human and non-human species. The aim of the former is to prolong survival while the latter is to improve quality of live and alleviate symptoms.

In canines, only 5/49 cases reported metastasis [45, 51, 54, 84, 99]. Lymph node metastasis occurred in two cases, while metastasis to the contralateral mammary gland, lungs, and pelvic region each occurred once. A rabbit mammary tumour had metastasised to the lungs, pleural lining, and liver [80]. Metastasis was not documented among rats, guinea pigs or the wolf. The follow-up period and prognosis varied between 2 weeks to 77 months. Felines had the highest proportion of tumour-related deaths. The survival of male animals with mammary tumours across all species is summarised in Table 4.

**Table 4:** Frequency of survival of male animals with breast cancer

Taxonomic group/Species	Case frequency			
	Alive at end of follow-up	Dead or euthanised at end of follow-up	Tumour related death (%)	Unreported
Canine (*n* = 84)	17 [49, 52–56]	26 [45–47, 50, 52–54, 85, 97]	2 (8%) [85, 97]	41 [48, 51, 54, 57, 65–70, 84, 86, 96, 97]
Feline (*n* = 43)	12 [61, 71]	17 [61, 62]	10 (59%) [61, 62]	14 [59–61]
Rodents (*n* = 10)	2 [72, 78]	2* [72, 79]	1 (50%) [79]	2 [74, 77]
Primates (*n* = 4)	1 [38]	3 [39, 40, 44]	0 (0%)	0
Rabbits (*n* = 1)	0	1^$^ [80]	1 (100%) [80]	0
Wolf (*n* = 1)	0	0	0 (0%)	1 [63]

^*^ Four additional diagnosed cases of mammary tumour in rodents had died or were euthanised prior to time of diagnosis and were not included

^$^ Rabbit died 6 months after due to severe respiratory distress likely due to pulmonary metastasis

## Omics Studies

Genomic and transcriptomic data are being generated for canines and are starting to dissect the molecular pathways of BC in canines [28, 100]. Somatic and germline variants of BRCA1 and BRCA2 have been determined in felines [101]. Many similarities have been found, e.g. the frequency of somatic PIK3CA mutations, PI3K-Akt pathway deregulation, germline genetic variants in BRCA1/2 and p53-signalling; nevertheless, there is a notable lack of males in these studies.

## Discussion

Male mammary tumours were predominantly reported in companion animals, likely due to the proximity to humans leading to a higher likelihood of incidental detection. Prevalence of mammary tumours in canines and felines may be attributed to their longer lifespan compared to rodents, increasing the probability of spontaneous oncogenic mutations. Tumour detection in all animals included in this study occurred past two-thirds of their lifespan, consistent with the older age distribution of BC in men [102].

Possible breed associations were identified with presentation in Spaniels, German Shephard, and Dachshunds. This is significant considering previous studies have noted a preponderance of mammary tumours in these breeds among female canines [58] and linked them to significant mutations found in human BC [103–105]. A study on Springer spaniels revealed a significant association between the development of mammary tumours and *BRCA1* and *BRCA2* polymorphisms, with 97% of diagnosed cases possessing these alleles [103]. In humans, *BRCA* mutations are present in 1–7% of the general population [106] and accounts for 5–10% of all breast cancer cases [106, 107]. However, BC in men is more typically associated with *BRCA2* mutations which is linked to a higher lifetime risk of developing BC compared to *BRCA1* carriers (1–5% versus 5–10%) [108].

BC classification in men is based on histological and molecular subtyping. Similar histological classifications exist between primates and humans and standardisation for canine and feline mammary tumour classification has been introduced [83]. Omics studies are still in their infancy for non-human species and males remain underrepresented. This is an area which deserves further study.

An unexpected finding was the identification of two less frequent manifestations of BC in humans, TNBC and IBC in male dogs. IBC closely mimicked the human disease in symptoms and histological features. Like male BC, IBC is also an uncommon form of BC accounting for just 2.5% of all cases in the US [109] with very few in men [110]. Both canine cases presented with painful erythematous mammary swellings alongside inflammatory infiltrate and dermal lymphatic vessels, like human cases [98, 109, 110]. Despite no reported metastasis, the short survival period in these canines mirrors the extremely poor prognosis observed in IBC in humans [98]. While 2 male canines diagnosed with TNBC had poor outcomes, dying within a few months of surgery or euthanised at time of diagnosis [45], a third remained recurrence-free for 6 months post-surgery [84].

While immunohistochemistry is used routinely in clinical workups for human BC for molecular classification and prognostication, this was less common in animal studies suggesting that veterinary medicine may prioritise tumour diagnosis and treatment over classification. Additionally, cost and availability of specialised testing in veterinary medicine, may further limit routine biomarker testing in animals. Indeed only 10% of the studies we examined reported on ER, PR, and HER2. This may change in the future, following a consensus statement from Brazil recommending a standard immunohistochemical panel for diagnosing mammary tumours in canines and felines, including ER, PR, Ki-67 and COX2 [111].

In contrast to BC management in men, where lumpectomy is uncommon due to limited breast tissue and adverse effects with adjuvant therapies, most animals in this study (where surgery was specified) underwent lumpectomies. An orangutan with a hormone-receptor positive tumour received hormonal adjuvant therapy at human dosages. Although its efficacy in animals remains unproven, the administration of anastrozole appeared to slow the lymph node enlargement, suggesting potential control of tumour growth. While the ATAC trial identified the benefits of anastrozole in the postmenopausal setting in women [112], its value in male BC has been insufficiently explored [113]. Nevertheless, the administration of adjuvant hormonal therapies in the orangutan demonstrates the potential for cross-species application of BC treatments.

The restrictive sample size across non-canine animals as well as insufficient and inconsistent reporting of the histology, biomarker expression, and prognosis of individual cases reduce the certainty of the conclusions made in comparison to human male BC. The relative recency of publication of the recommended guidelines for reporting of canine BC [111] limits the interpretation of reported histological features. Despite this, our work highlights pros, and cons of utilising animal models to understand human male BC. This needs redressing to enable full exploration of the One Health paradigm in rare cancers.

### Electronic Supplementary Material

Below is the link to the electronic supplementary material.

Supplementary Material 1

## Data Availability

No datasets were generated or analysed during the current study.
