# Supplementary material for: Exploring the One Health Paradigm in Male Breast Cancer
Source: J Mammary Gland Biol Neoplasia. 2024 Apr 4;29(1):8. doi: 10.1007/s10911-024-09560-6 (PMC10995048; doi:10.1007/s10911-024-09560-6)
Supplement: Supplementary file 1 — Supplementary Material 1 [file 10911_2024_9560_MOESM1_ESM.docx]

**Supplementary Tables**

**Table S1**

**Frequency of canine mammary tumours classified by the classification system proposed by Goldschmidt [55]**

| **Histological classification for canine mammary tumours** | **Number of cases reported** |
| --- | --- |
| Malignant Epithelial Neoplasms | 22 |
| Carcinoma in-situ | 1 |
| Carcinoma-simple (Tubulopapillary, cystic-papillary, cribriform) | 9 |
| Carcinoma–micropapillary invasive | 1 |
| Carcinoma–solid | 1 |
| Carcinoma–anaplastic | 2 |
| Carcinoma arising in a complex adenoma/mixed tumour | 2 |
| Carcinoma–complex type | 4 |
| Carcinoma–mixed type | 1 |
| Intraductal papillary carcinoma | 1 |
| Malignant Epithelial Neoplasms—Special Types | 5 |
| Squamous cell carcinoma | 1 |
| Adenosquamous carcinoma | 1 |
| Mucinous carcinoma | 1 |
| Inflammatory carcinoma | 2 |
| Carcinosarcoma: Malignant Mixed Mammary Tumour | 2 |
| **Total cases of malignant tumours:** | 29 |

**Table S2**

**Distribution of mammary tumour diameter across different species.**

| **Species (total tumours)** | **Mammary tumour diameter, frequency of tumours** | | |
| --- | --- | --- | --- |
|  | **< 2cm** | **2-3cm** | **> 3cm** |
| All species (n=67) | 23 | 17 | 27 |
| Canine (n=35) | 7 | 7 | 21 |
| Feline (n=18) | 11 | 4 | 3 |
| Rats (n=8) | 1 | 4 | 3 |
| Primates (n=4) | 2 | 2 | 0 |
| Wolf (n=1) | 1 | 0 | 0 |
| Rabbit (n=1) | 1 | 0 | 0 |
